# Ankyrin-1 Gene Exhibits Allelic Heterogeneity in Conferring Protection Against Malaria

**DOI:** 10.1534/g3.117.300079

**Published:** 2017-07-26

**Authors:** Hong Ming Huang, Denis C. Bauer, Patrick M. Lelliott, Matthew W. A. Dixon, Leann Tilley, Brendan J. McMorran, Simon J. Foote, Gaetan Burgio

**Affiliations:** * Department of Immunology and Infectious Disease, John Curtin School of Medical Research, Australian National University, Canberra 2601, Australia; † Commonwealth Scientific and Industrial Research Organisation, Sydney 2113, Australia; ‡ Immunology Frontier Research Center Research Building, Osaka University, Suita, 565-0871, Japan; § Department of Biochemistry and Molecular Biology, Bio21 Institute, Melbourne 3052, Australia

**Keywords:** ankyrin-1, malaria, erythrocyte cytoskeleton, allelic heterogeneity

## Abstract

Allelic heterogeneity is a common phenomenon where a gene exhibits a different phenotype depending on the nature of its genetic mutations. In the context of genes affecting malaria susceptibility, it allowed us to explore and understand the intricate host–parasite interactions during malaria infections. In this study, we described a gene encoding erythrocytic ankyrin-1 (*Ank-1*) which exhibits allelic-dependent heterogeneous phenotypes during malaria infections. We conducted an ENU mutagenesis screen on mice and identified two *Ank-1* mutations, one resulting in an amino acid substitution (MRI95845), and the other a truncated *Ank-1* protein (MRI96570). Both mutations caused hereditary spherocytosis-like phenotypes and confer differing protection against *Plasmodium chabaudi* infections. Upon further examination, the *Ank-1^(MRI96570)^* mutation was found to inhibit intraerythrocytic parasite maturation, whereas *Ank-1^(MRI95845)^* caused increased bystander erythrocyte clearance during infection. This is the first description of allelic heterogeneity in ankyrin-1 from the direct comparison between two *Ank-1* mutations. Despite the lack of direct evidence from population studies, this data further supported the protective roles of ankyrin-1 mutations in conferring malaria protection. This study also emphasized the importance of such phenomena in achieving a better understanding of host–parasite interactions, which could be the basis of future studies.

Malarial parasites have been coevolving with humans for thousands of years and have played a major role in shaping the human genome in malaria-endemic regions (Kwiatkowski 2005; Hedrick 2012). Indeed, many genetic polymorphisms were selected for as they provide significant host survival advantages during malaria infections (Kwiatkowski 2005; Williams 2006b), resulting in high frequencies of protective genetic mutations in malaria-endemic regions. The majority of these affect the red blood cells (RBCs), and hence the blood stage of malaria infections (Williams 2006a,b; Lell *et al.* 1999).

Interestingly, these genetic mutations or alleles often exhibit varying degrees of malaria protection even if they affect the same gene, which is influenced by the location and the severity of the mutations (Clark *et al.* 2009; Fry *et al.* 2009). This phenomenon, known as “allelic heterogeneity,” is characterized by multiple different phenotypes arising from mutations in a single gene. It has been described for certain genes affecting malaria susceptibility, which is reflected by their geographical distribution within malaria-endemic regions (Bauduer 2013). One of the most prominent examples of this is the G6PD deficiency disorder, which can arise from multiple mutations in the G6PD gene (Beutler *et al.* 1991; Clark *et al.* 2009). Many studies have explored the effectiveness of each mutation in protecting individuals from malaria, which corresponds to the distribution of each allele across the globe (Howes *et al.* 2013; Shah *et al.* 2016; Clarke *et al.* 2017). Another example is the β-globin gene, which is well known for its two malaria protective alleles, HbS and HbC in African populations (Kilian *et al.* 2015; May *et al.* 2007). HbC is restricted to West Africa, whereas HbS is widespread throughout Africa, which is thought to be linked to the effectiveness of each allele at conferring malaria resistance and their nonmalaria-associated morbidity (Kreuels *et al.* 2010; Gonçalves *et al.* 2016). Studies on these alleles would not only allow a better understanding of host–parasite interactions, but would also give us insights into the dynamics of population genetics in malaria-endemic regions (Bauduer 2013).

However, allelic heterogeneity can also complicate the characterization of the malaria-protective roles of certain genes, often resulting in conflicting evidence from various studies. One example of such polymorphisms is CD36 deficiency, which was originally thought to be protective against malaria, as evidenced by the positive selection in East Asian and African populations (Aitman *et al.* 2000; Curtis and Aster 1996; Urwijitaroon *et al.* 1995). While some studies reported increased malaria protection (Pain *et al.* 2001), others reported no significant associations (Amodu *et al.* 2005), or even increased susceptibility (Ayodo *et al.* 2007; Aitman *et al.* 2000). It is possible that these contradictive findings are due to confounding factors associated with allelic heterogeneity in CD36 deficiency (Fry *et al.* 2009). This further emphasizes the importance of taking allelic heterogeneity into consideration and to design better future studies involving host genetics in malaria, as well as various other infectious diseases.

In terms of malaria susceptibility, however, the allelic heterogeneity of genes affecting the RBC cytoskeleton is poorly understood. Many of the resulting genetic disorders are heterogeneous, such as hereditary spherocytosis (HS), which is characterized by the formation of “spherocytic” RBCs that exhibit reduced volume due to disruptions in the erythrocyte cytoskeletons. HS is caused by mutations in ankyrin, spectrins, band 3, and protein 4.2, with ankyrin mutations contributing to >50% of all HS cases (Gallagher and Forget 1998; Jarolim *et al.* 1996; Takaoka *et al.* 1994; Matsuda *et al.* 1995; Eber *et al.* 1996), where the severity depends greatly on the location and the nature of mutations (Gallagher 2005). However, the prevalence of HS in malaria-endemic regions is not well studied; only isolated cases were reported (Sangerman *et al.* 2008; Spector and Metz 1963; Hassan *et al.* 2009; Ustun *et al.* 2003). Nevertheless, *in vivo* and *in vitro* studies have repeatedly suggested an association of HS with increased malaria resistance, and several mechanisms have been proposed, although not all of them were consistent (Shear *et al.* 1991; Schulman *et al.* 1990; Rank *et al.* 2009; Greth *et al.* 2012). Based on these observations, we hypothesized that the inconsistencies in resistance mechanisms might be due to the allelic heterogeneity of genes associated with HS.

To explore this hypothesis, we examined mouse models carrying two novel N-ethyl-N-nitrosourea (ENU)-induced ankyrin mutations. These two mouse lines, *Ank-1^(MRI96570/+)^* and *Ank-1^(MRI95845/MRI95845)^*, displayed hematological and clinical features consistent with HS, and a marked resistance to infection by the murine malarial parasite, *Plasmodium chabaudi*. Analysis of the underlying mechanism of resistance to infection revealed both common and distinct features between the strains. RBCs from both mouse lines were similarly resistant to merozoite invasion. Although the *Ank-1^(MRI95845/MRI95845)^* erythrocytes were more rapidly cleared from circulation during an infection, an impairment in intraerythrocytic parasite maturation was observed in the infected *Ank-1^(MRI96570/+)^* erythrocytes. This study highlights the first direct examination of allelic heterogeneity of the *Ank-1* gene in the context of malaria resistance in mouse models.

## Materials and Methods

### Mice and ethics statement

All mice used in this study were housed with 12 hr light-dark cycles under constant temperature at 21°, with food and water available *ad libitum*. All procedures were performed according to the National Health and Medical Research Council Australian code of practice. Experiments were carried out under ethics agreement AEEC A2014/54, which was approved by the animal ethics committees of the Australian National University.

### ENU mutagenesis and dominant phenotype screening

SJL/J male mice were injected intraperitoneally with two doses of 100 mg/kg ENU (Sigma-Aldrich, St Louis, MO) at 1 wk intervals. The treated males (G0) were crossed to females from the isogenic background to produce the first generation progeny (G1). The 7-wk-old G1 progeny were bled and analyzed on an Advia 120 Automated Hematology Analyzer (Siemens, Berlin, Germany) to identify abnormal RBC count. A mouse carrying the MRI96570 or MRI95845 mutation was identified with an RBC “mean corpuscular volume” (MCV) value three SD lower than other G1 progeny. It was crossed with SJL/J mice to produce G2 progeny to test the heritability of the mutations and the dominant mode of inheritance. Mice that exhibited a low MCV (<48 fL) were selected for whole exome sequencing and genotyping.

### Whole exome sequencing

DNA from two G2 mice per strain carrying the abnormal RBC parameters (MCV < 48 fL) were extracted with the Qiagen DNeasy Blood and Tissue Kit (Qiagen, Venlo, Netherlands) for exome sequencing as previously described (Hortle *et al.* 2016). Briefly, 10 µg of DNA was prepared for exome enrichment with the Agilent SureSelect kit paired-end genomic library from Illumina (San Diego, CA), followed by high throughput sequencing using a HiSequation 2000 platform. The bioinformatics analysis was conducted according to the variant filtering method previously described by Bauer *et al.* (2015). Private variants that were shared between the two mutants but not with other SJL/J, C57BL/6 mice, excluding the common variants between other ENU mouse strains, were annotated using ANNOVAR (Wang *et al.* 2010). Private nonsynonymous exonic and intronic variants within 20 bp of the exon spicing sites were retained as potential candidate ENU mutations.

### PCR and Sanger sequencing

DNA from mutant mice was amplified through PCR with 35 cycles of 30 sec of 95° denaturation, 30 sec of 56–58° annealing, and 72° elongation for 40 sec. The primers used in the PCR are described below in Table 1 and Table 2. The PCR products were examined with agarose gel electrophoresis before being sent to the Australian Genome Research Facility in Melbourne, Australia, for Sanger sequencing. The logarithm of odds (LOD) score was calculated based on the number of mice that segregated with the candidate mutations.

**Table 1:** Primers for identifying the MRI95845 mutation

Amplicon	Forward	Reverse
*Snai2*	CATCTGCAGACCCACTCTGA	TGGTTGGTAAGCACATGAGAA
*Tbc1d23*	CACCCCCTTTTTGGTTTCTT	ACGTGCACATCGACTAACCA
*Pnpla6*	AGGCTGAGGAAGTGTGCCTA	AACTAGCTGGGCTTTGGTCA
*Zglp1*	CTGGCCTTTGACTTCTGACC	CCTCACAAGGTGGCTGTTTC
*Ank-1*	CTCCAAGTGAGAGGGTTTGC	GATGGCACACAGTCAGACCA

**Table 2:** Primers for identifying the MRI96570 mutation

Amplicon	Forward	Reverse
*Fat4*	CGCATCCCTTCATACAACCT	ACACCCCACTCACGTAGCTC
*Rhcg*	TGAGGAATGAGGGAGAAAGG	CCAATATGGCAGCCCTCTAA
*Plxnb3*	TACCCGATCAATCCAGAAGG	TTCTGAATGTGCAGGGTCAC
*Ank-1*	TGTGCAGGCATTTCTACATGA	ACTCTCTGGGTAGACCCCGT

### RBC osmotic fragility analysis

To assess the susceptibility of the RBC membrane to osmotic stress, 5 µl of mouse whole blood was diluted 100-fold with phosphate buffer (pH 7.4) containing 0–10 g/L of sodium chloride, and incubated for at least 10 min at room temperature. The cells were centrifuged at 800 × *g* for 3 min, and the supernatant, which contains free hemoglobin, was measured at 540 nm to assess the degree of hemolysis. The absorbance values were expressed as percentage of hemolysis, with hemolysis at 0 g/L sodium considered as 100% lysis.

### Erythrocyte lifetime assay

Each uninfected mouse was intravenously injected with 1 mg of EZ-link Sulfo-NHS-LC Biotin (Biotin) (Thermo Scientific, Waltham, MA) in mouse tonicity PBS (MT-PBS). 2 μl of blood was collected on 1, 7, 14, 21, and 28 d postinjection. Samples were stained and analyzed using a flow cytometer. The proportion of Biotin-labeled mature RBCs on day 1 was considered as the “starting point” of 100% of labeled cells. For subsequent time points, the remaining number of Biotin-labeled RBCs were expressed as a percentage of the starting number as an indication of the RBC turnover rate.

For infected mice, 1 mg of Biotin was injected intravenously as soon as parasitemia was detectable via flow cytometry (∼0.05–0.3%). Samples were collected daily and analyzed as above.

### Ektacytometry

10–15 μl of uninfected RBCs were first resuspended in 500 μl of prewarmed polyvinylpyrrolidone solution at a viscosity of 30 mPa/sec at 37° until needed. Samples were analyzed according to the manufacturer’s instructions with a RheoScan Ektacytometer (Rheo Meditech, Seoul, South Korea) and the elongation index measured across a range of pressures, from 0–20 Pa. Each sample was measured three times to account for technical variabilities. The values were normalized against the wild-type samples.

### In vitro spleen-retention assay

The RBC deformability was examined according to the protocol described previously by Deplaine *et al.* (2011), with modifications. Briefly, RBCs from each genotype of mice were stained with 10 µg/ml of either hydroxysulfosuccinimide Atto 633 (Atto 633) or hydroxysulfosuccinimide Atto 565 (Atto 565) (Sigma-Aldrich, St Louis, MO), followed by three washes with MTRC (154 mM NaCl, 5.6 mM KCl, 1 mM MgCl_2_, 2.2 mM CaCl_2_, 20 mM HEPES, 10 mM glucose, 4 mM EDTA, 0.5% BSA, pH 7.4, filter sterilized). The stained RBCs were mixed in equal proportion and diluted with unstained, wild-type RBCs to result in ∼10–20% of the total RBCs being labeled RBCs. The samples were further diluted to 1–2% hematocrit with MTRC, before passing through the filter bed. The prefiltered and postfiltered samples were analyzed on a BD LSRFortessa (BD Biosciences, Franklin Lakes, NJ) flow cytometer to determine the proportion being retained in the filter bed.

### Scanning electron microscopy

Scanning electron microscopy (SEM) was performed as described previously (Huang *et al.* 2016). Mouse blood was fixed overnight in 3% EM-grade glutaraldehyde (Sigma-Aldrich) at 4° immediately upon collection. The samples were washed with MT-PBS 3 times, 10 min each time. The cells were then adhered to the coverslips with 0.1% polyethylenimine (PEI) for 10 min, before washing with MT-PBS. The cells were then dried serially using 30, 50, 70, 80, 90, 100, and 100% ethanol, for 10 min each. The cells were then soaked in 1:1 ethanol: hexamethyldisilazane solution for 10 min, followed by two washes with 100% hexamethyldisilazane (Sigma-Aldrich), each for 10 min. The coverslips were then air dried overnight, coated with gold, and then examined under a JEOL JSM-6480LV scanning electron microscope.

### Quantitative PCR and cDNA sequencing

RNA was purified from embryonic livers of E14 embryos using Qiagen RNeasy kit (Qiagen), followed by cDNA synthesis using a Transcriptor High Fidelity cDNA Synthesis Kit (Roche, Basel, Switzerland), as described previously (Huang *et al.* 2016). Quantitative PCR was performed on the ViiA 7 Real-Time PCR System (Thermo Scientific). The ΔΔC_T_ method (Livak and Schmittgen 2001) was used to determine the cDNA levels of *Ank-1* and the housekeeping gene β-actin and was expressed as a fold-change of the mutants to the wild type. The primers used for the *Ank-1* gene spanned exon 2–4: *Ank-1*-F: 5′-TAACCAGAACGGGTTGAACG-3′; *Ank-1*-R: 5′-TGTTCCCCTTCTTGGTTGTC-3′; β-Actin-F: 5′-TTCTTTGCAGCTCCTTCGTTGCCG-3′; β-Actin-R: 5′-TGGATGCGTACGTACATGGCTGGG-3′.

### SDS-PAGE, Coomassie staining, and Western blot

The analysis of erythrocytic proteins was carried out as described previously (Huang *et al.* 2016). Briefly, RBC ghosts were prepared by lysing mouse RBCs with ice-cold 5 mM phosphate buffer (ph7.4) and centrifuging at 20,000 × *g* for 20 min, followed by removal of the supernatant, repeating this process until the supernatant became clear. The RBC ghosts or whole blood lysates were denatured in SDS-PAGE loading buffer (0.0625 M Tris, pH 6.8, 2% SDS, 10% glycerol, 0.1 M DTT, 0.01% bromophenol blue) at 95° for 5 min before loading onto a 4–15% Mini-PROTEAN TGX Precast Gels (Bio-Rad, Hercules, CA). The gels were then either stained with Coomassie Blue solution (45% v/v methanol, 7% v/v acetic acid, 0.25% w/v Brilliant Blue G) overnight or transferred to a nitrocellulose membrane. The Western blot was carried out using these primary antibodies: 1 μg/ml anti-alpha 1 spectrin (clone 17C7), 1 μg/ml anti-beta 1 spectrin (clone 4C3) (Abcam, Cambridge, United Kingdom), 0.5 μg/ml anti-GAPDH (clone 6C5) (Merck Millipore, Darmstadt, Germany), anti-N-terminal *Ank-1* “p89” at 1:1500 dilution, anti-band 3 at 1:4000 dilution, and anti-protein 4.2 at 1:2000 dilution (kind gifts from Connie Birkenmeier, Jackson Laboratory). Each primary antibody was detected with the appropriate horseradish peroxidase (HRP)-conjugated secondary antibody at 1:5000 dilution from 1 mg/ml stocks. The blots were visualized using ImageQuant LAS 4000 (GE Healthcare Life Sciences, Arlington Heights, IL), and quantified using ImageJ software (Schneider *et al.* 2012).

### Malaria infection

Malaria infections on mice were performed as described previously (Huang *et al.* 2016). 200 µl of thawed *P. chabaudi adami*-infected blood was injected into the intraperitoneal cavity of a C57BL/6 donor mouse. When the donor mouse reached 1–10% parasite load (parasitemia), blood was collected through cardiac puncture. The blood was diluted with Krebs’ buffered saline with 0.2% glucose, as described previously (Jarra and Brown 1985). Each experimental mouse was infected with 1 × 10^4^ parasites intraperitoneally. The parasitemia of these mice were monitored either using light microscopy or flow cytometry.

### Terminal deoxynucleotidyl transferase dUTP nick end labeling staining

The terminal deoxynucleotidyl transferase dUTP nick end labeling (TUNEL) assay was carried out as described previously (Huang *et al.* 2016), with slight modification. 3 µl of infected blood containing 1–10% parasitemia were collected during the trophozoite stage and fixed in 1:4-diluted BD Cytofix Fixation Buffer (BD Biosciences) for at least a day until they were needed. Each sample was then washed twice with MT-PBS, and adhered to a glass slide precoated with 0.1% PEI for 10 min at room temperature. The excess cells were washed and the glass slide was incubated overnight at room temperature with TUNEL labeling solution (1 mM cobalt chloride, 25 mM Tris-HCl, pH 6.6, 200 mM sodium cacodylate, 0.25 mg/ml BSA, 60 μM BrdUTP, 15U terminal transferase). The slides were washed three times, followed by staining with 50 µg/ml of anti-BrdU-FITC antibody (Novus Biologicals, Littleton, CO) in MT-PBT (MT-PBS, 0.5% BSA, 0.05% Triton X-100) for 1 hr. The slides were then washed three times with MT-PBS, mounted with SlowFade Gold Antifade Reagent with DAPI (Thermo Scientific), and sealed. When the slides were dried, they were examined using an Axioplan 2 fluorescence light microscope (Carl Zeiss, Oberkochen, Germany) using magnifications between 60 and 100×. At least 100 DAPI-positive cells were counted, and each was graded as either positive or negative for TUNEL staining as an indication of DNA fragmentation.

### In vivo erythrocyte tracking assays

The *in vivo* erythrocyte tracking (IVET) assay was carried out as previously described by Lelliott *et al.* (2014), (2015). Briefly, at least 1.5 ml whole blood was collected from mice of each genotype via cardiac puncture, followed by staining with either 10 µg/ml of Atto 633 or 125 µg/ml of Biotin (Thermo Scientific) for 45 min at room temperature, followed by washing two times with MT-PBS. The blood was mixed in two different dye combinations to correct for any dye effects. ∼1 × 10^9^ erythrocytes were injected intravenously into infected wild-type mice at 1–5% parasitemia during the schizogony stage. Blood samples were collected at various time points, from 30 min up to 36 hr after injection, and analyzed using flow cytometry. The ratio of infected, labeled erythrocytes was determined as an indication of the relative susceptibility of RBCs to *P. chabaudi* infections. The proportion of labeled blood populations was also tracked over time to determine the clearance of these RBCs from the circulation.

### Flow cytometry analysis of blood samples

For erythrocyte lifetime assays, 2 µl of whole blood samples was stained with 2 μg/ml streptavidin-PE-Cy7, 1 μg/ml anti-CD71-allophycocyanin (APC) (clone R17217), 1 μg/ml anti-CD45–APC eFluor 780 (clone 30-F11) (eBioscience, San Diego, CA), 4 µM Hoechst 33342 (Sigma-Aldrich), and 12 µM JC-1 (Thermo Scientific) in MTRC. The samples were washed once with MTRC and further stained with 2 μg/ml streptavidin-PE-Cy7 to capture all Biotin-labeled cells. Immediately prior to analyzing on the flow cytometer, 5 µl of 123count eBeads (eBioscience) was added to determine the relative levels of anemia.

For both malaria infections and the IVET assay, 2 µl of whole blood samples were stained with 2 μg/ml streptavidin-PE-Cy7 (only for experiments with biotinylated erythrocytes), 1 μg/ml anti-CD45-APC-eFluor 780 (clone 30-F11), 1 μg/ml anti-CD71 (TFR1)-PerCP-eFluor 710 (clone R17217) (eBioscience), 4 µM Hoechst 33342 (Sigma-Aldrich), and 12 µM JC-1 (Thermo Scientific) in MTRC. All samples analyzed through flow cytometry were performed on BD LSRFortessa (BD Biosciences), where 200,000–1,000,000 events were collected and visualized on FACSDiva and FlowJo software. RBCs were identified by gating on CD71-negative and CD45-negative populations, followed by gating on Atto-labeled and Biotin-labeled erythrocytes on appropriate channels (APC for Atto-633, PE for Atto-565, and PE-Cy7 for Biotin). The parasitemia of each labeled erythrocyte population was determined by gating on Hoechst 33342-positive and JC-1-positive populations.

### Statistical analysis

The statistical analysis was carried out as described in a previous study (Huang *et al.* 2016). Unless otherwise stated, all statistical analysis was carried out using unpaired two-tailed Student’s *t*-tests without corrections. In particular, the LOD score method coupled with Bonferroni correction was used to determine the causative mutation for MRI96570 and MRI95845, in which the significance threshold and the LOD score for each candidate mutation was calculated, and a LOD score above the threshold was considered statistically significant. The statistical tests for the fluorescence recovery after photobleaching (FRAP) assay, ektacytometry, RBC lifetime assay, and clearance assays were performed by first calculating the area under curves for each sample, followed by an unpaired Student’s *t*-test on pooled mice of each genotype. The statistical tests for *in vitro* spleen-retention assays were performed using a paired Student’s *t*-test, comparing mutant with wild-type RBC populations within the same sample. The statistical significance of the malaria survival was tested using the unpaired log-rank test on pooled mutant *vs.* wild-type mice. The statistical significance of *Plasmodium* infection was determined via the statmod software package for R (http://bioinf.wehi.edu.au/software/compareCurves) using the “compareGrowthCurves” function with 10,000 permutations, followed by adjustments for multiple testing. The statistical significance for the ratios of IVET assays was determined using the one sample *t*-test with a hypothetical mean of one.

### Data availability

The Supplemental Material, File S1, contains additional data described in this article: Table S1 in File S1 contains the candidate genes investigated in this study and their associated LOD scores; Table S2 in File S1 contains the data from the hematological screen using the ADVIA 120 hematology analyzer; Figure S1 in File S1 shows the sequencing results; Figure S2 in File S1 shows the RBC morphology under light microscopy and SEM; Figure S3 in File S1 shows the expression of various cytoskeletal proteins; and Figure S4 in File S1 indicates the parasitemia during IVET assays. Other raw data are available upon request.

## Results

### MRI96570 and MRI95845 carry mutations in Ank-1 gene

**Figure 1:**
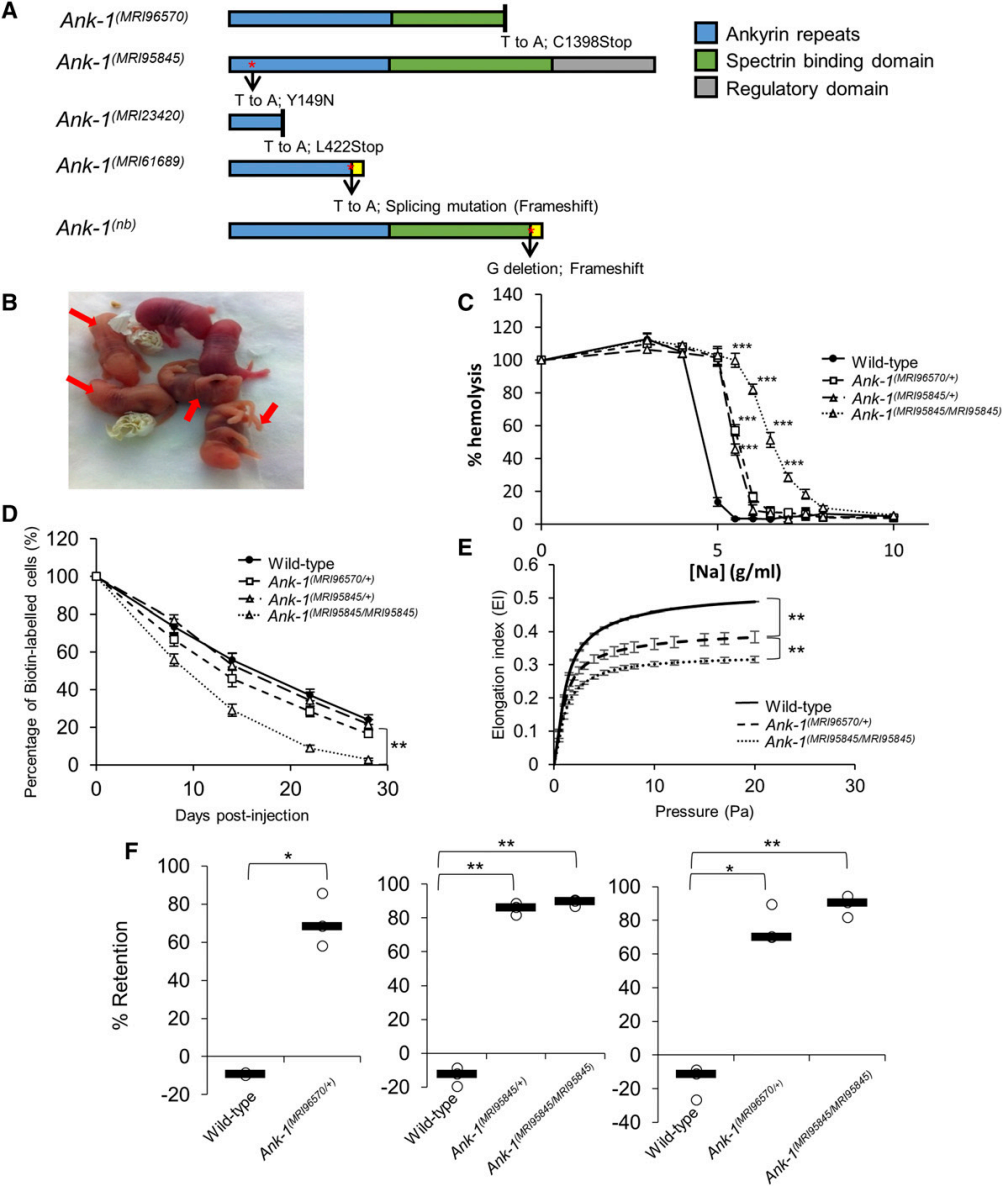
The mutations and phenotypes of *Ank-1^(MRI96570/+)^*, *Ank-1^(MRI95845/+)^*, and *Ank-1^(MRI95845/MRI95845)^* mice. (A) The location of ankyrin-1 mutations in *Ank-1^(MRI96570)^* and *Ank-1^(MRI95845)^* alleles and the predicted effects on ankyrin-1 protein, compared to the previously described *Ank-1^(MRI23420)^*, *Ank-1^(MRI61689)^*, and *Ank-1^(nb)^*. (B) The *Ank-1^(MRI96570/MRI96570)^* pups (indicated by ←) showed severe jaundice and died within 1 wk after birth. (C) The osmotic fragility of *Ank-1^(MRI96570/+)^*, *Ank-1^(MRI95845/+)^*, and *Ank-1^(MRI95845/MRI95845)^* erythrocytes in hypotonic solution from 0 to 10 g/L sodium (*n* = 5). (D) The RBC half-life of *Ank-1^(MRI96570/+)^*, *Ank-1^(MRI95845/+)^*, and *Ank-1^(MRI95845/MRI95845)^* mice (*n* = 5). (E) The elasticity of *Ank-1^(MRI96570/+)^* and *Ank-1^(MRI95845/MRI95845)^* RBCs under shear pressure as measured by ektacytometer (*n* = 3). (F) The proportion of retained *Ank-1^(MRI96570/+)^*, *Ank-1^(MRI95845/+)^*, and *Ank-1^(MRI95845/MRI95845)^* RBCs when passing through a layer of beads during the *in vitro* spleen retention assay (*n* = 3). All error bars indicate SEM. * *P* < 0.05, ** *P* < 0.01, *** *P* < 0.001.

ENU-treated SJL/J male mice were crossed with wild-type female to produce G1 progeny. The G1 progeny carrying heterozygous point mutations in their genome were then subjected to hematological screening to identify genes affecting RBC properties that might confer malaria protection. Heterozygous G1 mice MRI96570 and MRI95845 were identified from the ENU-dominant screen with MCV values three SD below the normal level of the respective parental line: 48.5 fL for MRI96570, and 50.6 fL for MRI95845, compared to the background of 55.1 ± 1.2 fL in the SJL/J inbred strain. These mice were crossed with wild type to produce G2 progeny, where approximately half exhibit low erythrocyte MCV values. Exome sequencing was carried out from two MRI96570 and MRI95845 G2 progeny showing a reduction of MCV to identify the causative genetic mutations. Unique variants shared between these mice were identified and listed in Table S1 in File S1. A mutation in the ankyrin-1 (*Ank-1*) gene was present in all the affected mice, and it cosegregated completely with the reduced MCV phenotype for over three generations of crosses. Sanger sequencing revealed a T to A transversion in exon 34 of the *Ank-1* gene for the MRI96570 strain, and a T to A transversion in exon 5 of the *Ank-1* gene for the MRI95845 strain (Figure S1 in File S1). These were predicted to cause a nonsense mutation at amino acid position 1398, located in the spectrin-binding domain for MRI96570 mice, and a substitution of tyrosine for asparagine at amino acid residue 149 in the fourth ankyrin repeat for MRI95845 mice (Figure 1A). MRI96570 and MRI95845 will be referred to as *Ank-1^(MRI96570)^* and *Ank-1^(MRI95845)^*, respectively, for the rest of the report.

### Both Ank-1^(MRI96570)^ and Ank-1^(MRI95845)^ exhibit HS-like phenotypes

Since ankyrin mutations are usually associated with HS, we examined both *Ank-1^(MRI96570)^* and *Ank-1^(MRI95845)^* mice in terms of their HS-like phenotypes. When two *Ank-1^(MRI96570/+)^* G2 progeny were intercrossed, *Ank-1^(MRI96570/MRI96570)^* mice were born with severe jaundice and died within several days of birth (Figure 1B), suggesting that homozygosity for the *Ank-1^(MRI96570)^* mutation caused lethal anemia. On the other hand, *Ank-1^(MRI95845/MRI95845)^* mice appeared healthy with a normal life span. Hematological analysis of these mice revealed a significant reduction in MCV and mean corpuscular hemoglobin, and increased red cell distribution width (Table S2 in File S1), indicating microcytosis and anisocytosis, which are the hallmarks for HS. When the RBCs were subjected to osmotic stress, RBCs from *Ank-1^(MRI96570/+)^*, *Ank-1^(MRI95845/+)^*, and *Ank-1^(MRI95845/MRI95845)^* mice exhibit significantly increased osmotic fragility compared to wild-type RBCs (Figure 1C). In particular, the sodium chloride concentration required to achieve 50% hemolysis is significantly higher (*P* < 0.001) for *Ank-1^(MRI96570/+)^* RBCs (5.6 g/L or 104 mM) and *Ank-1^(MRI95845/+)^* RBCs (5.4 g/L or 100 mM), compared to wild-type RBCs (4.6 g/L or 84 mM). The *Ank-1^(MRI95845/MRI95845)^* RBCs showed further susceptibility to osmotic stress, with 50% hemolysis at a sodium chloride concentration of ∼6.5 g/L (121 mM).

We predicted that the mutant RBCs have a shorter half-life, which is also one of the symptoms of HS. Therefore, RBC half-life was determined by biotinylating mouse RBCs *in situ* and tracking the proportion of biotinylated RBCs in circulation over time. As shown in Figure 1D, erythrocytes from *Ank-1^(MRI95845/MRI95845)^* have a significantly shorter half-life of ∼9.5 d as opposed to the 16 d of wild-type erythrocytes (*P* = 0.008), but no significant difference was observed for erythrocytes from heterozygous mice [*P* = 0.09 for *Ank-1^(MRI96570/+)^* mice and *P* = 0.08 for *Ank-1^(MRI95845/+)^* mice]. The morphology of these RBCs were examined under light and SEM (Figure S2 in File S1). *Ank-1^(MRI96570/+)^* and *Ank-1^(MRI95845/+)^* mice exhibited a slight reduction in RBC size, while *Ank-1^(MRI95845/MRI95845)^* mice had smaller acanthocytic RBCs and displayed anisocytosis. On the other hand, blood smears obtained from jaundiced *Ank-1^(MRI96570/MRI96570)^* pups showed reticulocytosis, fragmented RBCs, and severe anisocytosis.

Another feature of HS is reduced RBC deformability, which was examined using two different analytical techniques: ektacytometry and an *in vitro* spleen-retention assay. Ektacytometry measures the flexibility of RBCs when subjected to shear pressure, and expresses as an elongation index, which indicates the deformability of RBCs. The *Ank-1^(MRI96570/+)^* RBCs showed a reduced elongation index compared to wild type, with *Ank-1^(MRI95845/MRI95845)^* RBCs showing further reduction in elongation index, indicating significant reduction in RBC deformability (Figure 1E). In addition, the *in vitro* “spleen mimic” retention assay was performed by passing the erythrocytes through layers of microbeads of varying sizes, modeling *in vivo* splenic filtration. RBC deformability was assessed by the ability of RBCs to pass through the bead layer. Figure 1F showed three independent measurements of RBC deformability using the splenic-retention assay, comparing wild-type, *Ank-1^(MRI96570/+)^*, *Ank-1^(MRI95845/+)^*, and *Ank-1^(MRI95845/MRI95845)^* RBCs. An ∼70% increased retention for *Ank-1^(MRI96570/+)^* RBCs was observed compared to wild type, whereas erythrocytes of *Ank-1^(MRI95845/+)^* and *Ank-1^(MRI95845/MRI95845)^* mice showed 86 and 90% increased RBC retention compared to wild type, respectively. However, no significant difference was observed between *Ank-1^(MRI96570/+)^* and *Ank-1^(MRI95845/MRI95845)^* erythrocytes.

The expression levels of ankyrin and other RBC membrane proteins were also examined (Figure S3 in File S1). A significant reduction of *Ank-1* mRNA levels was observed in *Ank-1^(MRI96570/+)^*, *Ank-1^(MRI95845/+)^*, *Ank-1^(MRI96570/MRI96570)^*, and *Ank-1^(MRI95845/MRI95845)^* embryonic livers (Figure S3A in File S1). However, Coomassie staining and Western blotting of the RBC membrane fractions did not show a significant difference in ANK-1 levels between wild-type, *Ank-1^(MRI96570/+)^*, and *Ank-1^(MRI95845/MRI95845)^* erythrocytes (*P* = 0.3) (Figure S3, B–D, in File S1) using an anti-ANK-1 antibody (p89) specifically targeting the N-terminal region of ANK-1 protein (Greth *et al.* 2012). The predicted truncated ANK-1*^(MRI96570/+)^* form (160 kDa) was also not evidenced, suggesting degradation of truncated protein. The levels of other cytoskeletal proteins were also examined to account for possible disruptions to interactions with binding partners of ankyrin-1. However, no difference was observed for band 3, α-, and β-spectrin, whereas a significantly lower protein 4.2 level was observed only in *Ank-1^(MRI95845/MRI95845)^* erythrocytes (Figure S3D in File S1).

### Ank-1^(MRI96570)^ and Ank-1^(MRI95845)^ confer protection against P. chabaudi infection

**Figure 2:**
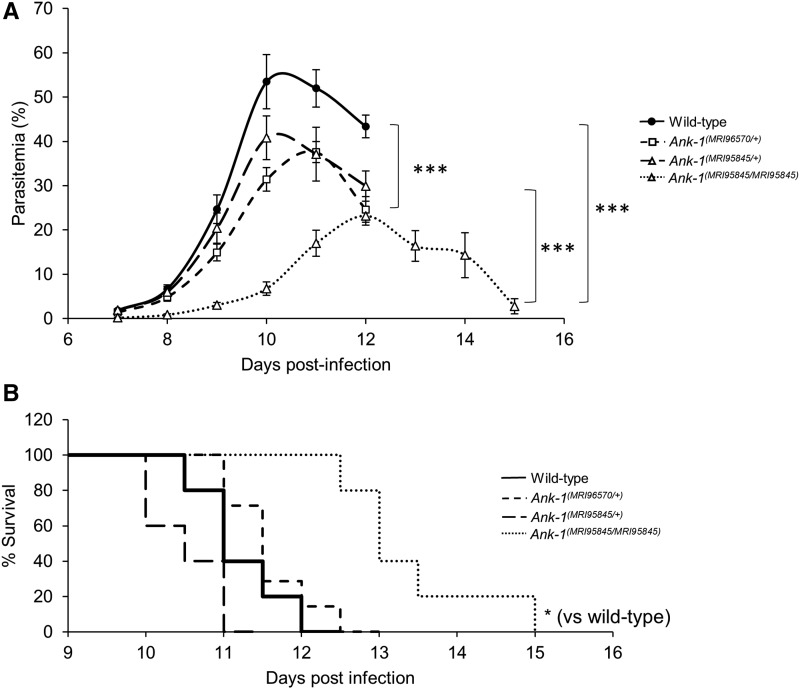
The parasitemia and survival of *Ank-1^(MRI96570/+)^*, *Ank-1^(MRI95845/+)^*, and *Ank-1^(MRI95845/MRI95845)^* mice during *P. chabaudi* infection. (A) The parasite load and (B) survival rate of *Ank-1^(MRI96570/+)^*, *Ank-1^(MRI95845/+)^*, and *Ank-1^(MRI95845/MRI95845)^* mice from two independent experiments, starting from day 7 to day 15 postinfection when challenged with 1 × 10^4^ parasite intraperitoneally injected, as determined by light microscopy (*n* = 9–13). Error bars indicate SEM. * *P* < 0.05, *** *P* < 0.001.

We proposed that mice carrying these mutations have reduced susceptibility to malaria infection, which we examined by injecting a lethal dose of *P. chabaudi* and recording the percentage of parasitized RBCs (parasitemia). As shown in Figure 2A, *Ank-1^(MRI96570/+)^* and *Ank-1^(MRI95845/+)^* mice showed significant reduction in peak parasitemia of ∼15–20%, while *Ank-1^(MRI95845/MRI95845)^* mice showed ∼30% reduction in peak parasitemia compared to wild type. *Ank-1^(MRI95845/MRI95845)^* mice also showed a 2-d delay in parasitemia, peaking on day 12 postinfection rather than day 10 as with wild type. *Ank-1^(MRI95845/MRI95845)^* mice also exhibited a significantly higher survival rate compared to wild type during *P. chabaudi* infection, but no significant difference was observed for *Ank-1^(MRI96570/+)^* and *Ank-1^(MRI95845/+)^* mice compared to wild type (Figure 2B). Overall, these results suggested that both *Ank-1^(MRI96570/+)^* and *Ank-1^(MRI95845/+)^* mice showed moderate resistance, whereas *Ank-1^(MRI95845/MRI95845)^* mice exhibited significant resistance toward *P. chabaudi* infection relative to wild-type mice.

From these results, we further investigated and compared the possible mechanisms of resistance mediated by *Ank-1^(MRI96570)^* and *Ank-1^(MRI95845)^* mutations. We examined three important determinants of parasite growth and survival within the host. First, we studied the ability of the parasite to survive within these erythrocytes, since ankyrin-1 mutations have previously been implicated in impairing parasite intraerythrocytic maturation (Greth *et al.* 2012). Second, the erythrocyte invasion was assessed, since the mutations disrupt erythrocyte cytoskeletal structure, which is important for facilitating efficient erythrocyte invasion (Chishti *et al.* 1996). Third, the mutations might result in an improved detection of parasitized RBCs, thus enhancing their removal from circulation during malaria infection. Since *Ank-1^(MRI96570/+)^* and *Ank-1^(MRI95845/MRI95845)^* mice exhibited differences in malaria resistance, we hypothesized that they mediate malaria resistance through different pathways.

### Ank-1^(MRI96570/+)^ and Ank-1^(MRI95845/MRI95845)^ erythrocytes are resistant to merozoite invasion

**Figure 3:**
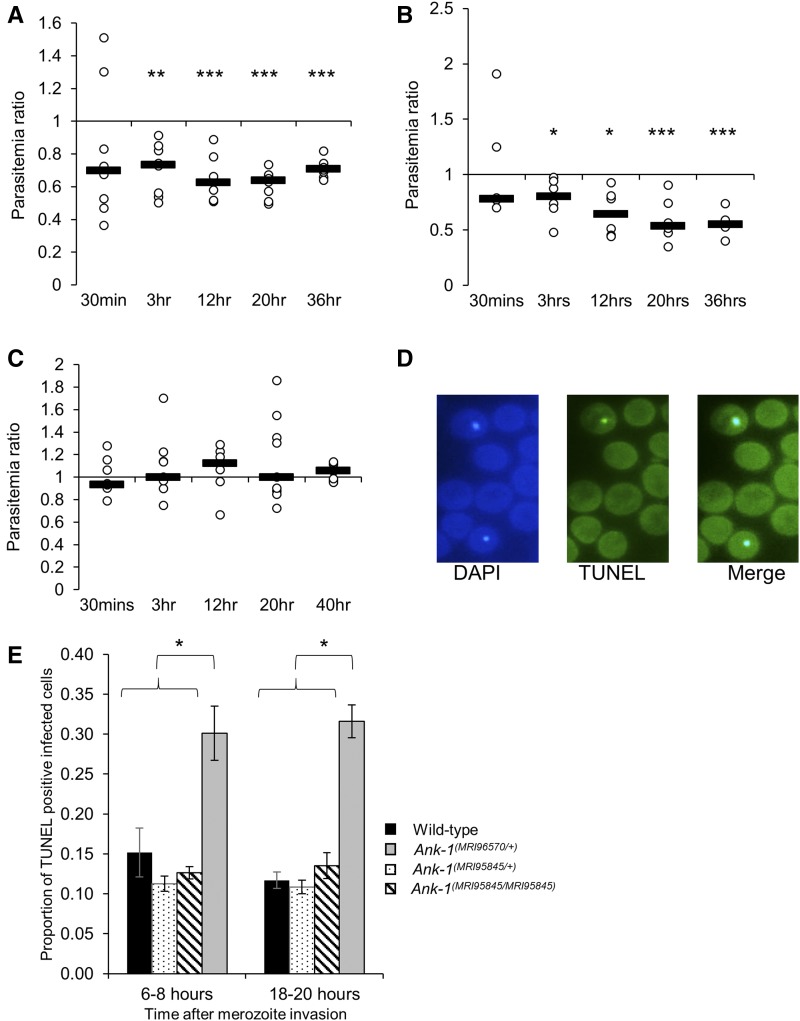
The parasite invasion and intraerythrocytic growth as indicated via IVET and TUNEL assay. The relative invasion efficiency into *Ank-1^(MRI96570/+)^* and *Ank-1^(MRI95845/MRI95845)^* erythrocytes was examined through IVET assay, where parasitemia ratio was calculated from parasite load of either (A) *Ank-1^(MRI96570/+)^* to wild-type, (B) *Ank-1^(MRI95845/MRI95845)^* to wild-type, or (C) *Ank-1^(MRI96570/+)^* to *Ank-1^(MRI95845/MRI95845)^* erythrocytes (*n* = 5–7 per group). (D) The parasite growth inhibition was determined via TUNEL assay on infected RBCs (DAPI positive) as an indicator of apoptotic and necrotic parasites. (E) The proportion of TUNEL-positive infected RBCs was counted for *Ank-1^(MRI96570/+)^*, *Ank-1^(MRI95845/+)^*, and *Ank-1^(MRI95845/MRI95845)^* mice at 1–5% parasitemia at ring stage (6–8 hr) and late trophozoite (18–20 hr) stage (*n* = 4). Error bars indicates SEM. * *P* < 0.05, ** *P* < 0.01, *** *P* < 0.001.

First, the ability of parasites to invade erythrocytes was assessed via an IVET assay. Labeled RBCs from either wild-type, *Ank-1^(MRI96570/+)^*, or *Ank-1^(MRI95845/MRI95845)^* mice were injected into infected wild-type mice of 1–10% parasitemia during late schizogony stage, and the parasitemia of each genotype was monitored over 36–40 hr to indicate relative invasion rates. The initial invasion period was expected at 30 min to 3 hr postinjection, and the results were expressed as a ratio of parasitized RBCs of either *Ank-1^(MRI96570/+)^* to wild type (Figure 3A), *Ank-1^(MRI95845/MRI95845)^* to wild type (Figure 3B), or *Ank-1^(MRI96570/+)^* to *Ank-1^(MRI95845/MRI95845)^* (Figure 3C). From Figure 3, A and B, *Ank-1^(MRI96570/+)^* and *Ank-1^(MRI95845/MRI95845)^* erythrocytes were less parasitized compared to wild type [0.6–0.7 for *Ank-1^(MRI96570/+)^* to wild type, *P* < 0.001; and 0.55–0.8 for *Ank-1^(MRI95845/MRI95845)^* to wild type, *P* < 0.001] from 3 hr up to 36 hr postinjection, indicating both *Ank-1^(MRI96570/+)^* and *Ank-1^(MRI95845/MRI95845)^* erythrocytes were more resistant to parasite invasion than wild type. However, no significant differences in parasitemia ratios were observed at the 30 min time point. Furthermore, when the invasion rate of both *Ank-1^(MRI96570/+)^* and *Ank-1^(MRI95845/MRI95845)^* erythrocytes were compared in infected wild-type mice (Figure 3C), no significant difference in parasitemia ratio was observed, suggesting a similar invasion rate between the two mutant erythrocytes.

### Ank-1^(MRI96570/+)^ erythrocytes impair parasite maturation

Second, the parasite intraerythrocytic maturation was determined through a TUNEL assay, which allows the detection of fragmented DNA in RBCs as an indication of dying parasites (Figure 3D) (McMorran *et al.* 2009). Samples were collected from infected mice at 1–10% parasitemia at both young ring stage and late trophozoite stage, and the proportion of TUNEL-positive infected RBCs were measured. As seen from Figure 3E, more TUNEL-positive parasites were observed within *Ank-1^(MRI96570/+)^* erythrocytes, in both ring (30.1 ± 3.4% compared to 15.2 ± 3.1% of wild type) and trophozoite stage (30.8 ± 3.8% compared to 11.7 ± 1.0% of wild type), whereas no differences were observed for *Ank-1^(MRI95845/+)^* and *Ank-1^(MRI95845/MRI95845)^* erythrocytes. This result suggested that the growth of parasites within *Ank-1^(MRI96570/+)^* erythrocytes was impaired, but was normal in *Ank-1^(MRI95845/+)^* and *Ank-1^(MRI95845/MRI95845)^* erythrocytes. This also indicates that *Ank-1^(MRI96570)^* disrupts parasite maturation, whereas *Ank-1^(MRI95845)^* seems to support normal parasite growth.

### Ank-1^(MRI95845/MRI95845)^ erythrocytes are more likely to be cleared during malaria infections, partially via splenic filtration

**Figure 4:**
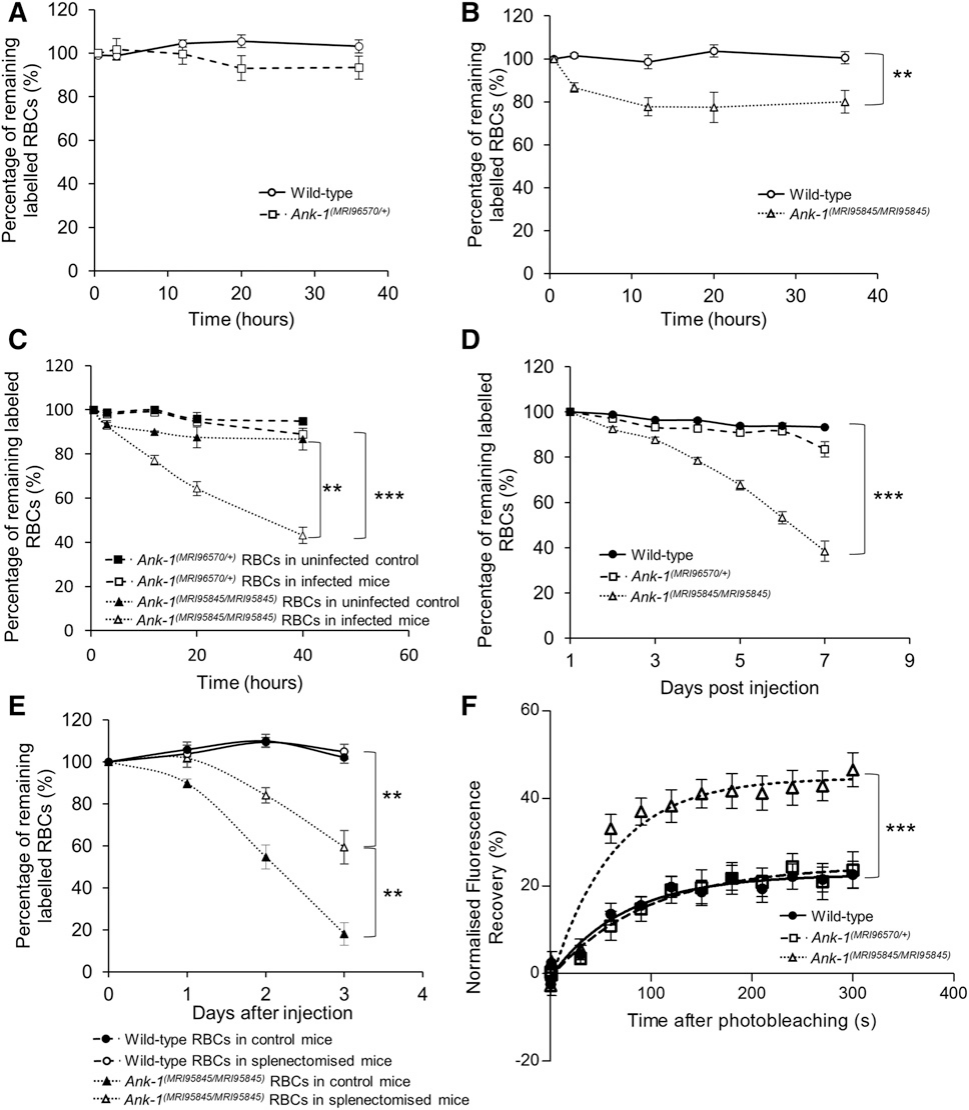
The band 3 mobility and clearance of wild-type, *Ank-1^(MRI96570/+)^*, and *Ank-1^(MRI95845/MRI95845)^* erythrocytes. The remaining percentage of labeled RBCs was monitored during the course of IVET assays, comparing between (A) wild-type and *Ank-1^(MRI96570/+)^* erythrocytes, (B) wild-type and *Ank-1^(MRI95845/MRI95845)^* erythrocytes, and (C) *Ank-1^(MRI96570/+)^* and *Ank-1^(MRI95845/MRI95845)^* erythrocytes (*n* = 5–7). (D) The half-life of wild-type, *Ank-1^(MRI96570/+)^*, and *Ank-1^(MRI95845/MRI95845)^* erythrocytes during malaria infection as determined by biotinylation of RBCs when parasites were detectable (*n* = 6–7). (E) The clearance of wild-type and *Ank-1^(MRI95845/MRI95845)^* erythrocytes in splenectomized and nonsplenectomized mice infected with *P. chabaudi* over 3 d starting from 1% parasitemia (*n* = 6). (F) The band 3 mobility on RBC membrane was measured using FRAP, showing the recovery rate of fluorescence as a result of band 3 migration to the bleach spot (*n* = 9–21). Error bars indicate SEM. ** *P* < 0.01, *** *P* < 0.001.

The proportions of labeled erythrocytes were also monitored during the IVET assays to compare the relative loss of the two labeled RBC populations as an indicator of RBC clearance during malaria infection. No significant reduction in *Ank-1^(MRI96570/+)^* erythrocyte numbers was observed during the IVET assay compared to wild type (Figure 4A). In contrast, the number of labeled *Ank-1^(MRI95845/MRI95845)^* erythrocytes decreased significantly compared to wild-type and *Ank-1^(MRI96570/+)^* erythrocytes (Figure 4, B and C), with ∼20 and 50% reduction, respectively. However, the parasitemia measurements during the IVET assays were ∼2% to 16–30% (Figure S4, A–B in File S1), which did not correlate with the reduction of labeled *Ank-1^(MRI95845/MRI95845)^* erythrocytes. This suggested an increased bystander clearance rather than clearance of infected *Ank-1^(MRI95845/MRI95845)^* RBCs. To further verify this observation, the infected mice from each genotype were injected with Biotin and the biotinylated RBC half-life was examined. As shown in Figure 4D, the *Ank-1^(MRI96570/+)^* mice exhibited no significant reduction in RBC numbers, whereas *Ank-1^(MRI95845/MRI95845)^* mice were found to have a significantly shorter half-life of ∼6 d, which did not correlate with the parasitemia curve (Figure S4C in File S1). This observation of shorter RBC half-life in infected *Ank-1^(MRI95845/MRI95845)^* mice is consistent with the increased *Ank-1^(MRI95845/MRI95845)^* erythrocyte clearance as shown in IVET assays.

We proposed that the spleen played a major role in mediating this bystander clearance. Therefore, we infected splenectomized mice with *P. chabaudi* and infused them with labeled wild-type and *Ank-1^(MRI95845/MRI95845)^* erythrocytes, the proportions of which were monitored over time. As shown in Figure 4E, *Ank-1^(MRI95845/MRI95845)^* erythrocyte numbers are approximately twofold higher (*P* < 0.01) in splenectomized mice compared to nonsplenectomized mice. This suggests that the spleen is a major contributor toward *Ank-1^(MRI95845/MRI95845)^* erythrocyte clearance, although the clearance was not completely abrogated in the absence of the spleen.

### Increased band 3 mobility in Ank-1^(MRI95845/MRI95845)^ erythrocytes as a likely mechanism for increased clearance

We hypothesized that this increase in RBC clearance is likely due to changes to the cytoskeletal structure of *Ank-1^(MRI95845/MRI95845)^* RBCs. To examine our hypothesis, we investigated the band 3 mobility within the RBC membrane as an indicator of the integrity of vertical linkages in the RBC cytoskeleton (Kodippili *et al.* 2009; Cho *et al.* 1998). We fluorescently labeled erythrocytic band 3 with eosin-5′-maleimide and performed FRAP on erythrocytes, which involves photobleaching a spot on the RBC surface with a pulse from a high-powered laser, followed by a fluorescence recovery period where the intensity was recorded over 5 min as a marker of mobility. *Ank-1^(MRI95845/MRI95845)^* RBCs were found to have significantly higher fluorescence recovery compared to wild-type and *Ank-1^(MRI96570/+)^* RBCs (Figure 4F), which suggests a higher band 3 mobility in *Ank-1^(MRI95845/MRI95845)^* erythrocytes, possibly due to an increased amount of band 3 that was not associated with the RBC cytoskeleton as a result of disrupted ankyrin binding to band 3 on the RBC surface.

## Discussion

### Ank-1 gene displayed allele-dependent heterogeneous phenotypes during malaria infections

Similar to HS in human populations, ankyrin mutations in mice also exhibit differences in clinical symptoms depending on the mutations. As shown in this study, homozygosity for the MRI96570 mutation is lethal and MRI95845 homozygotes appeared healthy; whereas both *Ank-1^(MRI96570/+)^* mice and *Ank-1^(MRI95845/+)^* mice exhibited HS-phenotypes with similar severity. While both mutations also conferred malaria protection and appeared to impair parasite invasion, they also showed some remarkable differences in mediating this resistance. Parasites in *Ank-1^(MRI96570/+)^* erythrocytes were more likely to be TUNEL positive, indicating impaired intraerythrocytic maturation; whereas *Ank-1^(MRI95845/MRI95845)^* erythrocytes were more likely to be removed from circulation and possibly had an increased turnover rate.

These findings were not exclusive to the two *Ank-1* mice described in this study. In fact, previous studies on other *Ank-1* mice also exhibit similar mechanisms of resistance. Notably, similar to *Ank-1^(MRI96570/+)^*, *Ank-1^(MRI23420/+)^* (Greth *et al.* 2012) and *Ank-1^(nb/nb)^* mice (Shear *et al.* 1991) were both reported to affect the parasite survival within the defective RBCs. These mutations resulted in truncated protein, therefore, it is possible that the loss of C-terminal ANK-1 protein might be important for growth. However, *Ank-1^(MRI61689/+)^* mice, which were also predicted to give rise to truncated protein, were found to exhibit increased RBC bystander clearance but no intraerythrocytic growth impairment (Huang *et al.* 2016), similar to the *Ank-1^(MRI95845/MRI95845)^* mice in this study.

Taking these findings together, it would seem that although these mutations reside in different parts of the ankyrin-1 gene, they all resulted in an HS-like phenotype. However, they exert different effects on the parasite survival depending on the nature of the mutation. In particular, nonsense mutations [*Ank-1^(nb)^*, *Ank-1^(MRI23420)^*, *Ank-1^(MRI96570)^*], with the exception of *Ank-1^(MRI61689)^*, impair parasite growth. On the other hand, the only described missense mutation, *Ank-1^(MRI95845)^*, increases the bystander RBC clearance. It is interesting to note that although the *Ank-1^(MRI61689)^* mutation was predicted to produce a truncated protein, a full-length alternative spliced transcript with a skipped exon was also found (Huang *et al.* 2016); possibly indicating that *Ank-1^(MRI61689)^* did not behave as a null mutation, consequently exhibiting different mechanisms of malaria resistance compared to other nonsense mutations. Therefore, it is proposed that the presence of full length, or at least functional, ANK-1 protein might be an important factor in determining the detrimental effects on malaria parasites.

However, without detailed examination of the RBC cytoskeletal structure, it is challenging to speculate the exact mechanisms of malaria resistance in these mutations. Nevertheless, this is the first direct report of such allelic heterogeneity described in *in vivo* malaria mouse models, highlighting the complexity underlying the genetic resistance to malaria, which is likely to correlate with human populations.

### Allelic heterogeneity of Ank-1 and its association with malaria

Due to the lack of large-scale studies on the HS prevalence in malaria-endemic regions, ankyrin-1 mutations have not been associated with malaria protection. Although HS prevalence is more well characterized in nonmalarial regions such as in Northern European and Japanese populations, with a prevalence of ∼1 in 2000 (Godal and Heisto 1981; Eber *et al.* 1992; Yawata *et al.* 2000), one study proposed an increased HS incidence in Algeria of ∼1 in 1000 (Zerhouni *et al.* 1991), raising the possibility of positive selection of HS by malarial parasites. However, as a result of the extreme allelic heterogeneity of HS-causing genes, many alleles do not reach sufficient frequencies (Kavallaris *et al.* 2012) or achieve consistent symptoms (Shah and Vega 2004) to be easily associated with malaria protection. In addition, technical difficulties (Sangerman *et al.* 2008), confounding factors from large genetic variation in African populations (Malaria Genomic Epidemiology Network 2008), as well as poor diagnostics and health systems (Malaria Genomic Epidemiology Network 2008) pose significant challenges for dissecting the connection between HS and malaria. Furthermore, the varying allele frequency in African populations might introduce epistasis effects, possibly masking the genotype–phenotype association typically observed in other populations (Greene *et al.* 2009). With the development of more advanced technologies and better characterization of the genetic structure of African populations, further studies into the association of HS and malaria could potentially yield beneficial insights into the coevolutionary relationships between humans and *Plasmodium*.

Nonetheless, previous *in vivo* studies have indicated that *Ank-1* mutations affect merozoite invasion and maturation (Shear *et al.* 1991; Greth *et al.* 2012), both of which were also demonstrated in this study. However, this study also describes for the first time the direct *in vivo* observation of different mutations in the *Ank-1* gene mediating two distinct, independent mechanisms of malaria resistance, where one impairs parasite maturation and the other increases RBC clearance. Ankyrin is one of the key proteins involved in RBC remodeling by parasites (Maier *et al.* 2009; Zuccala and Baum 2011; Hanspal *et al.* 2002) and maintaining the native structure of the RBC cytoskeleton (Miraglia del Giudice *et al.* 1994; Gallagher 2005). It is possible that this allelic heterogeneity is due to the effect each mutation has on the integrity of the RBC cytoskeletal structure, as evidenced by the significantly increased band 3 mobility caused by the *Ank-1^(MRI95845)^*, but not *Ank-1^(MRI96570)^*, mutation (Figure 4F). This suggests that mutations at different locations of the ankyrin-1 protein might have different effects on the RBCs, consequently impacting the ability of parasites to invade and grow, which could be the basis for further studies, while also taking into account potential confounding factors due to differences in genetic background.

### Similarities of allelic heterogeneity in Ank-1 and other malaria susceptibility genes

As evidenced from this study, the protective effect of the *Ank-1* gene against malaria is dependent on the nature and the location of mutations within the gene. Similarly, this allelic heterogeneity is also observed in other malaria susceptibility genes in human populations. For instance, although many G6PD deficiency-causing alleles have been implicated with malaria protection (Ruwende and Hill 1998; Mason *et al.* 2007), the protective effects are often debated, with many studies reporting increased or no protection for individuals with certain alleles of G6PD deficiency (Manjurano *et al.* 2015; Ruwende *et al.* 1995; Guindo *et al.* 2007; Sirugo *et al.* 2014; Toure *et al.* 2012; Martin *et al.* 1979). This is thought to be due to the phenotypic complexity of G6PD deficiency associated with malaria susceptibility (Clark *et al.* 2009). Indeed, various G6PD alleles have been shown to cause a reduction in G6PD activity with differing severity, and were proposed to correlate with the malaria protection they conferred (Manjurano *et al.* 2015). More recently, Clarke and colleagues proposed that reduced G6PD activity is associated with a lower risk of cerebral malaria, but in exchange for a higher risk of malarial anemia (Clarke *et al.* 2017), suggesting a delicate balance underlying the high frequency of G6PD polymorphism of populations in malaria-endemic regions. Similarly, *Ank-1* mutations described in this study, as well as other previous mouse studies (Greth *et al.* 2012; Rank *et al.* 2009; Shear *et al.* 1991), exhibit variability in malaria resistance, most likely as the result of allelic heterogeneity.

The heterogeneity in malaria resistance mechanisms of the *Ank-1* gene as observed in this study is comparable to the two prevalent alleles of the β-globin gene, HbS and HbC, which result from amino acid substitution at position six from glutamate to either valine or lysine, respectively. These two mutations exhibit some similarities in mediating malaria resistance, including impaired parasite growth (Fairhurst *et al.* 2003; Cyrklaff *et al.* 2011), reduced cytoadherence (Carlson *et al.* 1994; Fairhurst *et al.* 2012, 2005), and increased erythrocyte clearance (Ayi *et al.* 2004). However, HbS erythrocytes were found to be more resistant to all forms of malaria, whereas HbC erythrocytes appeared to be protective against cerebral malaria (Kreuels *et al.* 2010). This difference in malaria protection was proposed to correlate with distribution of HbS and HbC in Africa (Gonçalves *et al.* 2016), further emphasizing the importance of allelic heterogeneity in understanding host–parasite interactions.

In conclusion, we have reported a novel observation where the *Ank-1* gene exhibits phenotypic heterogeneity in malaria resistance mechanisms, either by impairing intraerythrocytic parasite growth or by promoting RBC clearance. This study also highlighted that the allelic heterogeneity in relation to malaria resistance is not exclusive to G6PD deficiency, and it could also be much more common than we expected. Further studies should extend the understanding of the effects of various *Ank-1* mutations on the development of intraerythrocytic parasites, as well as the association of HS with malaria in human populations. This could provide new insights into the intricate relationships between RBC cytoskeletal structure and parasite interactions.

## Supplementary Material

Supplemental material is available online at www.g3journal.org/lookup/suppl/doi:10.1534/g3.117.300079/-/DC1.
