# Supplementary material for: Ankyrin-1 Gene Exhibits Allelic Heterogeneity in Conferring Protection Against Malaria
Source: G3 (Bethesda). 2017 Jul 26;7(9):3133–44. doi: 10.1534/g3.117.300079 (PMC5592938; doi:10.1534/g3.117.300079)
Supplement: Supplementary file 1 [file 3133FileS1.pdf]

Table S1

Table S1. The candidate genes for MRI96570 and MRI95845 mutations.

A)

Chromosome	Gene name	Location	Reference base	Variant base	Number of mutant mice with mutation	LOD score (Threshold: 1.9)
3	*Fat4*	38888347	T	A	0/10	-2.81
7	*Rhcg*	79601661	T	C	0/10	-2.81
8	*Ank1*	23119400	T	A	10/10	2.81
X	*Plxnb3*	73763183	G	T	0/10	-2.81

B)

Chromosome	Gene name	Location	Reference base	Variant base	Number of mutant mice with mutation	LOD score (Threshold: 2.0)
8	*Ank1*	23085597	T	A	10/10	2.81
8	*Pnpla6*	3531116	G	A	8/10	1.24
9	*Zglp1*	21062907	G	A	0/10	-2.81
16	*Snai2*	14708259	A	C	0/10	-2.81
16	*Tbc1d23*	57191544	T	C	0/10	-2.81

Variants from exome sequencing were filtered to exclude strain-specific variants and variants found in other ENU-induced mice. Variants that were shared between the two mice carrying MRI96570 mutation or MRI95845 mutation are shown in (A) and (B), respectively. For each mutation, the candidate genes were Sanger sequenced in affected mice to determine the correlation between the genetic mutations and the phenotype by calculating the LOD score. LOD Threshold = 1.9 for MRI96570, 2.0 for MRI95845 (n= 10).

Figure S1

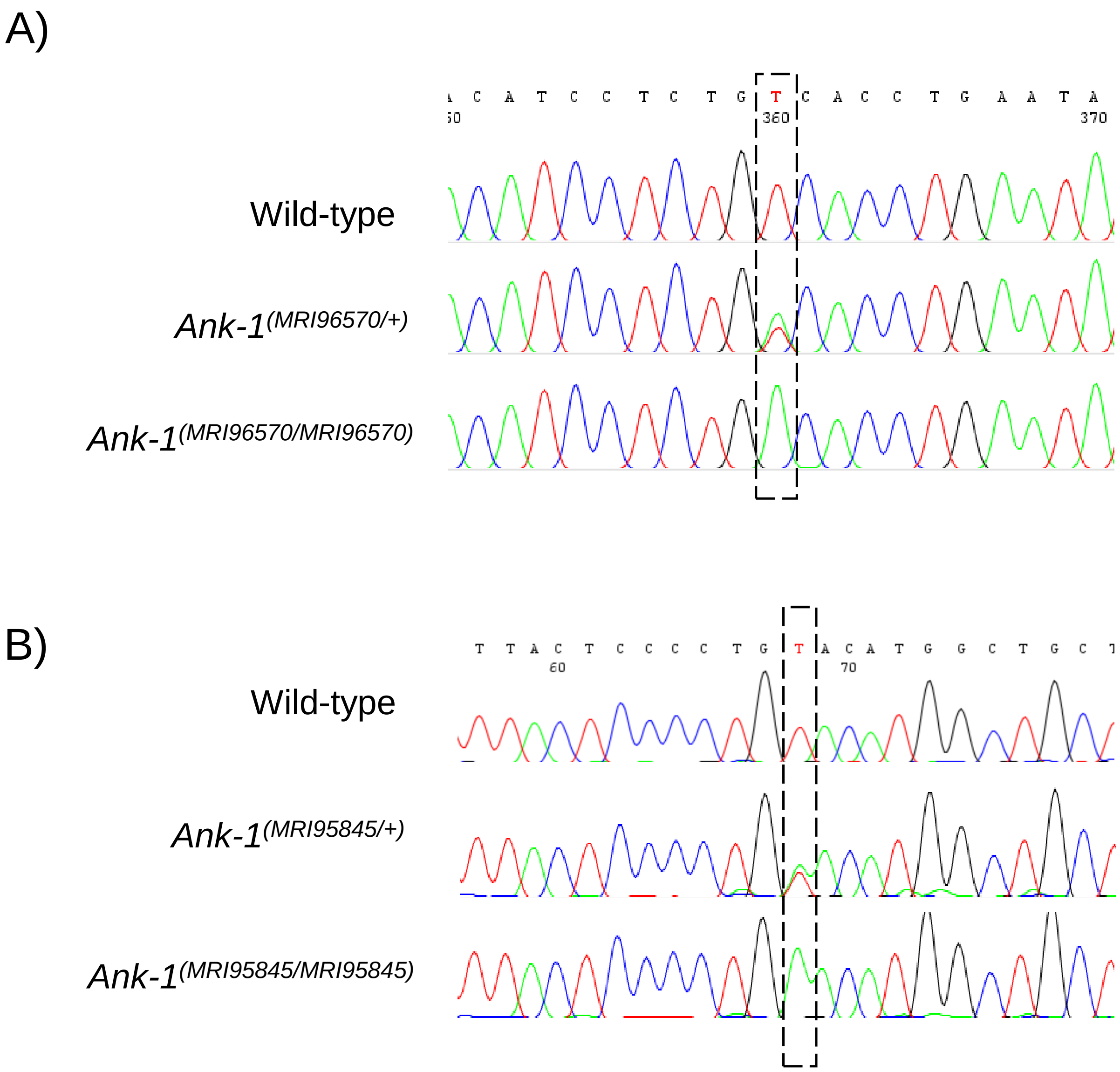

Figure S1. The location of *Ank-1*$^{(MRI96570)}$ and *Ank-1*$^{(MRI95845)}$ mutation. (A) Sanger sequencing of mice carrying *Ank-1*$^{(MRI96570)}$ revealed a T to A transversion in exon 34 of *Ank-1* gene, which is predicted to induce a premature stop codon. (B) Mice carrying *Ank-1*$^{(MRI95845)}$ mutation were found to have a T to A transversion in exon 5 of *Ank-1* gene, which is predicted to cause a missense mutation from tyrosine to asparagine at residue 149.

Table S2

Table S2. The complete blood count of *Ank-1*$^{(MRI96570/+)}$, *Ank-1*$^{(MRI95845/+)}$ and *Ank-1*$^{(MRI95845/MRI95845)}$ mice.

	Wild type	*Ank-1*$^{(MRI96570/+)}$	*Ank-1*$^{(MRI95845/+)}$	*Ank-1*$^{(MRI95845/MRI95845)}$
**WBC (x10^6 /ml)**	11.3±0.3	11.2±0.4	11.9±0.8	12.1±0.8
**RBC (x10^9 /ml)**	10.1±0.1	11.0±0.1***	10.7±0.1***	10.9±0.1***
**HGB (g/L)**	150.5±0.8	150.7±1.1	148.8±1.9	142.6±1.7*
**MCV (fL)**	52.3±0.2	46.9±0.2***	46.6±0.2***	43.0±0.1*** ^
**MCH (pg)**	14.9±0.1	13.7±0.1***	13.9±0.1***	13.1±0.2*** ^
**MCHC (g/L)**	285.3±1.2	290.3±1.8	298.3±2.4	294.7±4.0
**RDW (%)**	14.5±0.1	15.3±0.1***	16.1±0.3***	18.4±0.2*** ^
**PLT (x10^6 /ml)**	1036±31	1051±34	992±51	1026±53
**Retics (%)**	2.23±0.26	3.34±0.42	3.33±0.46	5.68±0.43***

The blood parameters were obtained from a haematological analyser ADVIA 120. WBC = white blood cell count; RBC = red blood cell count; HGB = total haemoglobin; MCV = mean corpuscular volume; MCH = mean corpuscular haemoglobin; MCHC = mean corpuscular haemoglobin concentration; RDW = red cell distribution width; PLT = platelet counts; Retics = percentage of reticulocytes, n=23-50. Bonferroni adjusted significance threshold = 0.001852, * P< 0.001, ** P< 1x10^{-5} compared to wild-type mice; whereas ^ P<0.001 compared to *Ank-1*$^{(MRI95845/+)}$ mice.

Figure S2

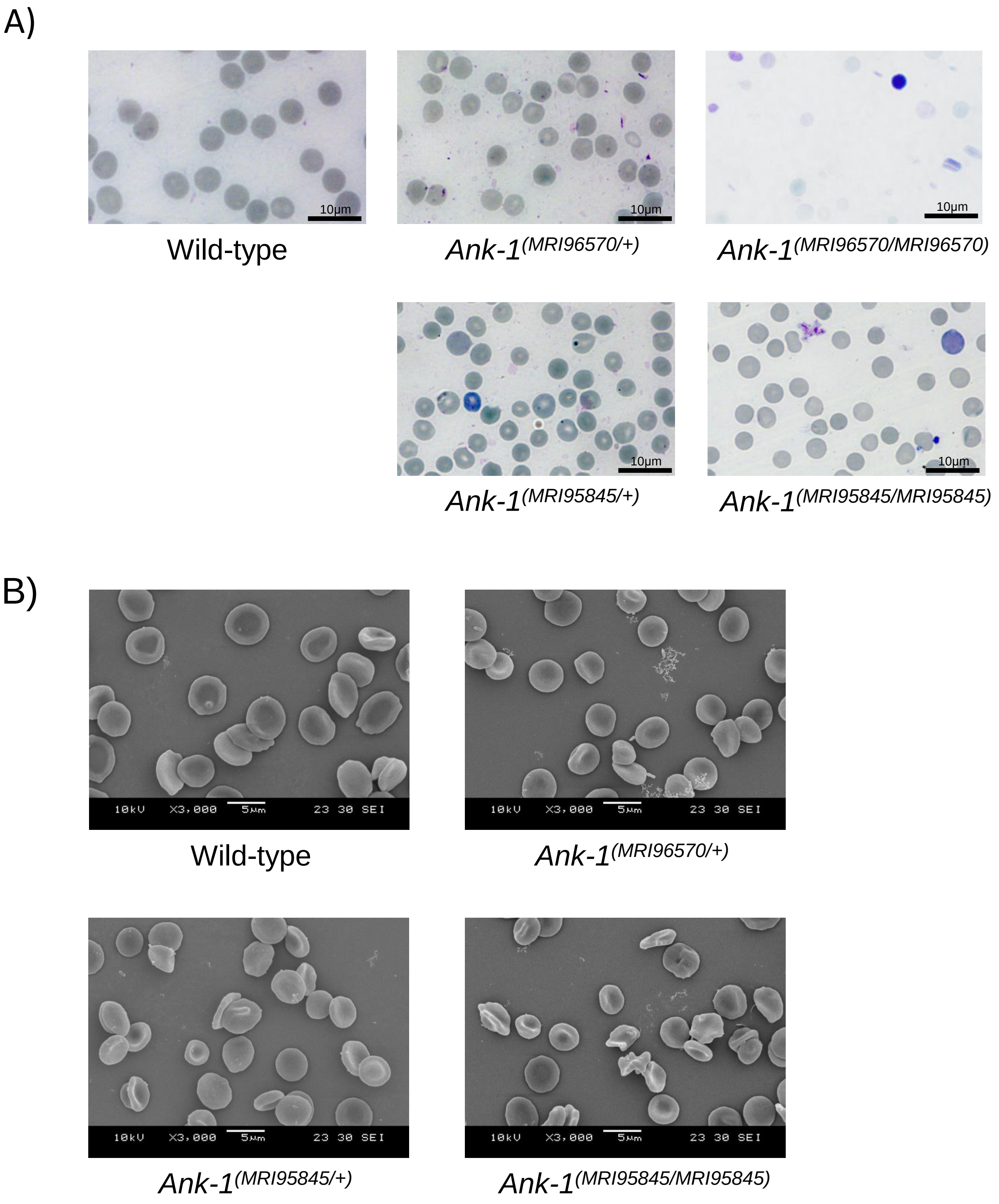

Figure S2. The RBC morphology of mice carrying *Ank-1*$^{(MRI96570)}$ or *Ank-1*$^{(MRI95845)}$ mutation. (A) Giemsa-stained blood smears as examined under light microscope at 1000x magnification. (B) Scanning electron microscopic images showing the RBC shape of *Ank-1*$^{(MRI96570/+)}$, *Ank-1*$^{(MRI95845/+)}$ and *Ank-1*$^{(MRI95845/MRI95845)}$ mice.

Figure S3

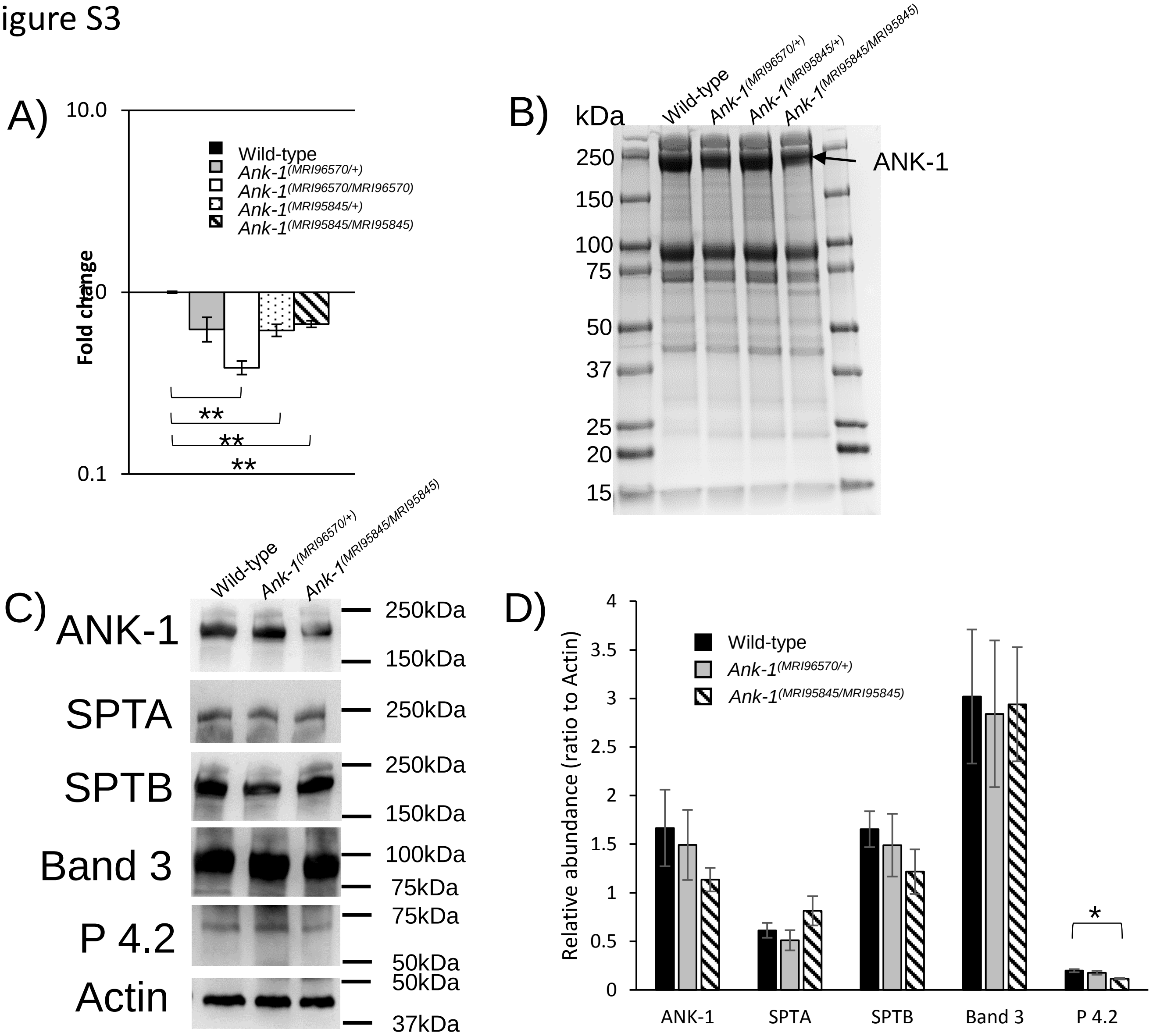

Figure S3. The expression of *Ank-1* and other RBC cytoskeletal proteins in mice carrying *Ank-1*$^{(MRI96570)}$ or *Ank-1*$^{(MRI95845)}$ mutation. (A) Quantitative PCR was carried out on E14 embryonic livers to examine *Ank-1* expression levels (n=3). The abundance of ANK-1 and other RBC cytoskeletal protein levels of *Ank-1*$^{(MRI96570/+)}$, *Ank-1*$^{(MRI95845/+)}$ and *Ank-1*$^{(MRI95845/MRI95845)}$ mice as examined via (B) Coomassie staining and (C) Western blotting on membrane of mature RBCs. (D) The relative abundance of various cytoskeletal protein levels calculated from western blots (n=3). SPTA = α-spectrin, SPTB = β-spectrin, P 4.2 = Protein 4.2. * P<0.05, ** P<0.01. Error bars indicate SEM.

Figure S4

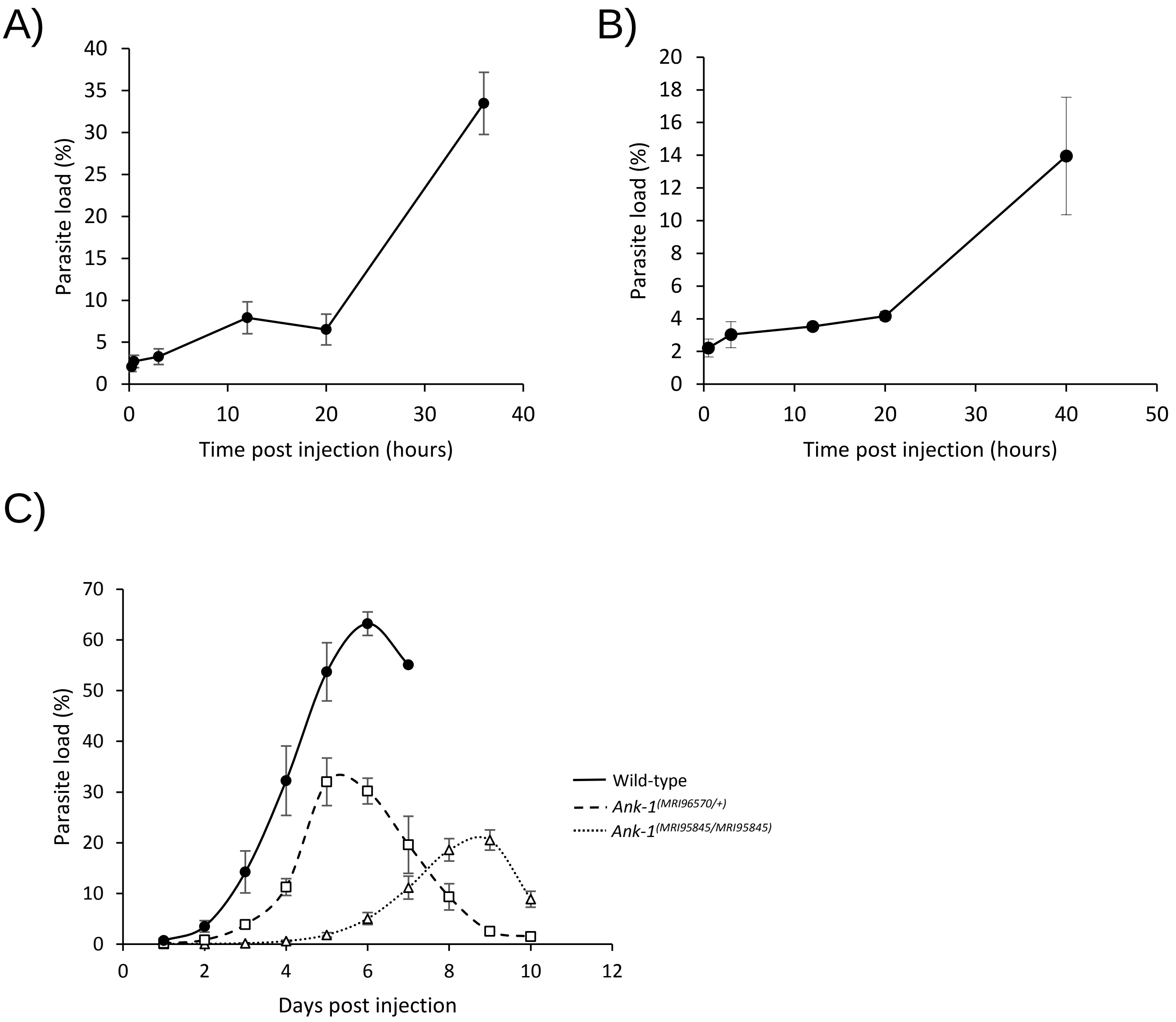

Figure S4. The parasitemia of the host mice during IVET assays and half-life assay. The parasitemia curve of the host mice during IVET assays, when comparing (A) wild-type with *Ank-1$^{(MRI95845/MRI95845)}$* erythrocytes, and (B) *Ank-1$^{(MRI96570/+)}$* with *Ank-1$^{(MRI95845/MRI95845)}$* erythrocytes (n=5-7). (C) The parasitemia curve of wild-type, *Ank-1$^{(MRI96570/+)}$* and *Ank-1$^{(MRI95845/MRI95845)}$* mice during RBC half-life assay (n=6-7).
